# Elusive cranial lesions severely afflicting young endangered Patagonian huemul deer

**DOI:** 10.1186/s13104-018-3755-1

**Published:** 2018-09-03

**Authors:** Werner T. Flueck

**Affiliations:** 1 Swiss Tropical and Public Health Institute, University Basel, Socinstrasse 57, 4051 Basel, Switzerland; 2 National Council of Scientific and Technological Research (CONICET), Buenos Aires, Argentina; 3 Argentine National Park Administration, C.C. 592, 8400 Bariloche, Argentina

**Keywords:** Huemul, *Hippocamelus bisulcus*, Osteopathology, Fenestration, Dehiscence, Acute periodontitis, Parodontitis, Clinical evaluation, Migration

## Abstract

**Objectives:**

Most subpopulations of endangered huemul deer (*Hippocamelus bisulcus*) fail to recover, frequently due to osteopathology. Equivalent pathology was detected only postmortem in an additional deer 365 km further north, stressing the need to improve clinical evaluations of live huemul.

**Results:**

Captured on a farm and attended by authorities in charge of huemul, the deer was considered apt for relocation and release. Delays with attendance and lack of reversal drugs resulted in his death. The subsequent necropsy revealed severe osteopathology particularly in mandibles and maxillae. Such disease in another southern population affected 57+ % among dead adults, and 86% among live adults. The present case stems from a new subpopulation, isolated 365 km further north. Such severe pathology demands that individuals be rehabilitated, especially relevant with severely endangered species, because liberations will cause premature death and loss of reproductive lifetime. Live huemul must be examined utmost professionally especially regarding this pathophysiognomy. This incidence represents the typical situation of extant huemul, being displaced from their traditional migratory behavior to utilize fertile low-elevation habitat. This young male may have been dispersing, but reaching valleys usually leads to death due to locally intense anthropogenic activities.

**Electronic supplementary material:**

The online version of this article (10.1186/s13104-018-3755-1) contains supplementary material, which is available to authorized users.

## Introduction

Patagonian huemul (*Hippocamelus bisulcus*), endemic cervid of southern Latin America, have been considered endangered for many decades due to tremendous post-Columbian declines in numbers and area of occupancy [1–3]. Later accounts of huemul stem exclusively from interior Andes where climatic extremes, steep topography, and closed vegetation delayed or prevented human colonization. Scientific interest in Argentina started in mid-1980s, and conservation efforts have been directed at the estimated 350–500 huemul remaining in some 50 subpopulations, spread along 1850 km of Andes [4]. There are no documented subpopulation recoveries, instead several have vanished or declined, even in protected areas. Furthermore, with rare species occurring mainly in remote refuges, reliable data is difficult to secure. Given the scarcity of substantiated knowledge about diseases affecting extant populations, skeletal remains found 1993–2007 provided initial insights about bone disease and its potential contribution to morbidity. Osteopathic prevalency among adults was 57+ %; affected individuals exhibited 63% mandibular, 100% maxillary, and 78% appendicular lesions. These lesions are the most parsimonious explanation for the young average age (3.1 years) and absent population recovery [2].

However, a most pivotal fact relates to severe absence of relevant diagnostic information based on in vivo examinations. The first-ever Argentine buck was only recently caught, examined and marked (08/2017), and Web-of-Science (04/2017) reveals only two articles about clinical observations in huemul (Chile): about external parasites [5], and about melanic skin fibromas [6]. This paper describes capture, clinical examination, death and necropsy of a huemul buck. It aims at amending discussions about prevalent occurrences of osteopathology in huemul with relevant new insight regarding the urgency of professional in vivo examinations, and its application for subsequent decisions and procedures. It also lends strong support for the causal relationship between restrained population dynamics and thus lack of recovery, and population health.

## Main text

### Methods

The capture occurred in the El Manso valley floor (436 m elevation, Argentina) located between mountains with elevations 1800 m above this valley floor, whereas the carcass was unearthed at 41°33′S, 71°28′W. Photographs and reports served to describe general circumstances, and necropsy procedures [7, 8] also aimed at recovering a maximum of tissues.

### Results

#### Case presentation

This huemul was cornered by dogs on a small farm, intercepted by chance by local people (20:30 h, 18/3/2016) who fastened it with ropes to a fence post to protect it from dogs, and immediately sought assistance from wildlife authorities. The area is known to have few remaining huemul in remote and surrounding higher areas, but it had been uncommon that huemul reached this valley for 6+ decades according to residents [9].

**Fig. 1:**
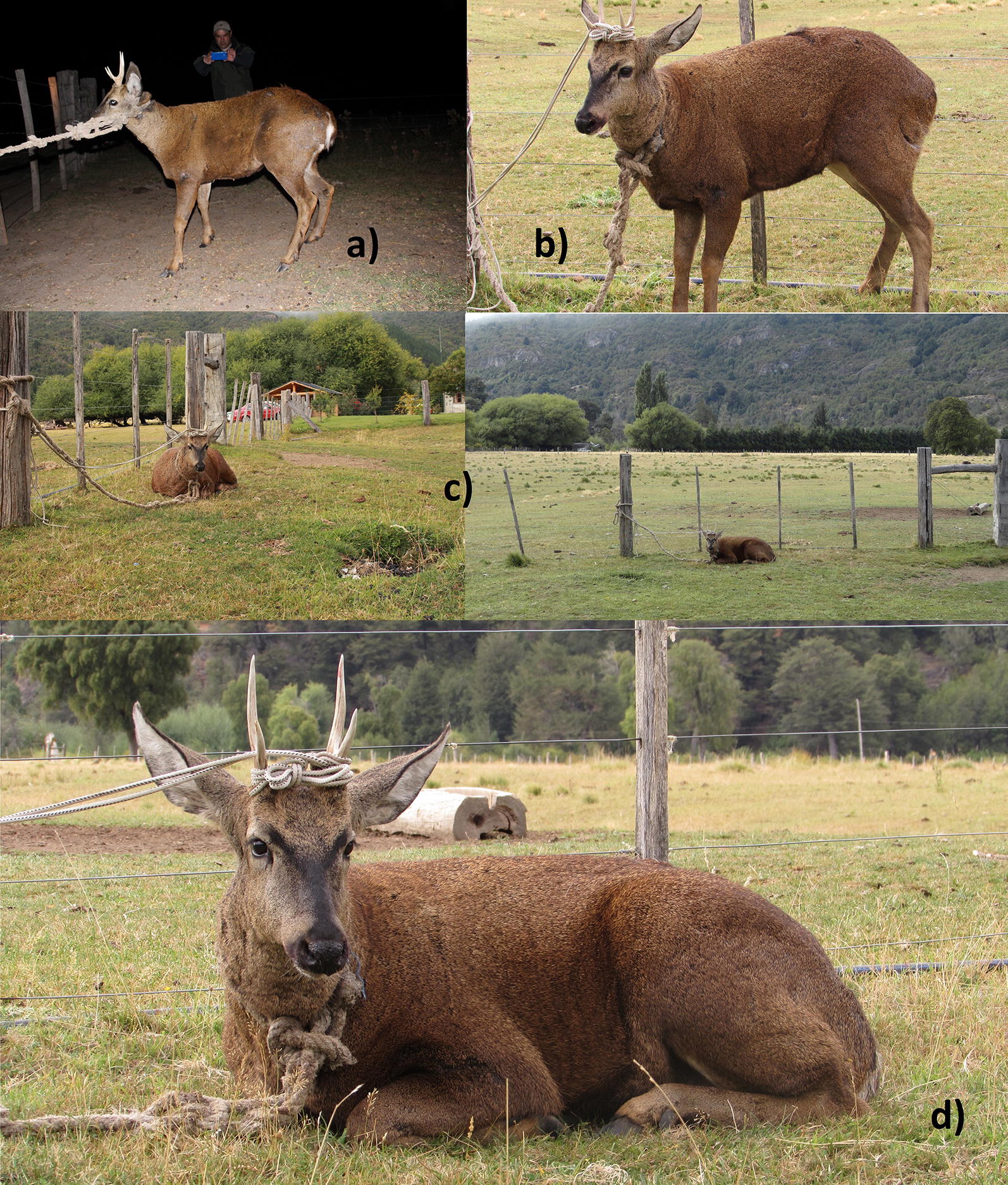
**a** The huemul buck after being restrained with a rope, approximately at 20:30 h, 18 March 2016; **b** the buck in the same spot about 14 h later; **c** the buck then bedded down, with view to SW (left) and E (right); **d** the bedded buck 15 h after having been roped

This captured male huemul appeared to adapt to the situation rather than resist, based on not trying to pull off the rope or jump into the fencing, and he even bedded down repeatedly (Fig. 1). Rather than expressing stress, this behavior concurs with consistent past descriptions of their initial curiosity and apparent fearlessness [10], or the recent experience of pre-capture interactions between several humans and 6 wild huemul ranging from 32 to 73 min and still allowing approaching them to within 10 m [11]. In the present case however, after extended delays, the authorities in charge of the National Huemul Program arrived the following day and > 16 h post-capture, with the aim to translocate the buck by 6.5 km before release. After sourcing and eventually receiving xylazine from a ranch 70 km further south, the huemul was sedated even 19 h after capture. After thorough examinations and morphometric measurements, he was translocated to be released, as no problems were encountered justifying treatment procedures or a temporary recovery in a rehabilitation center.

Due to absent reversal drugs, recovery was anticipated to occur via metabolic breakdown, but the huemul unfortunately died 23 h post-capture. Although apparently calm initially, this wild huemul eventually experienced likely elevated stress having been tied up for 19 h in a large open pasture near housing. Under such circumstances, using xylazine without reversal poses high risks, esteemed to have caused death, also when considering the good physical condition found postmortem. No blood nor tissues were collected, the carcass left on-site, although later, other people decided to bury it.

#### Necropsy

Fourteen weeks postmortem, the permit was finally received allowing to unearth the carcass for necropsy which, however, did reveal health issues. Being early autumn, the general body condition was good, based on presence of 25% omental fat [12], and 8 mm sternal fat deposits [13]. Pathological anomalies include various vertebral spines grown asymmetrically, and a perforation (fenestration, D = 8 mm) in the center of one scapula, both of which are very thin-boned. Tooth eruption pattern and wear, stage of cranial sutures, and antler size indicate that the age was 2.5 years.

**Fig. 2:**
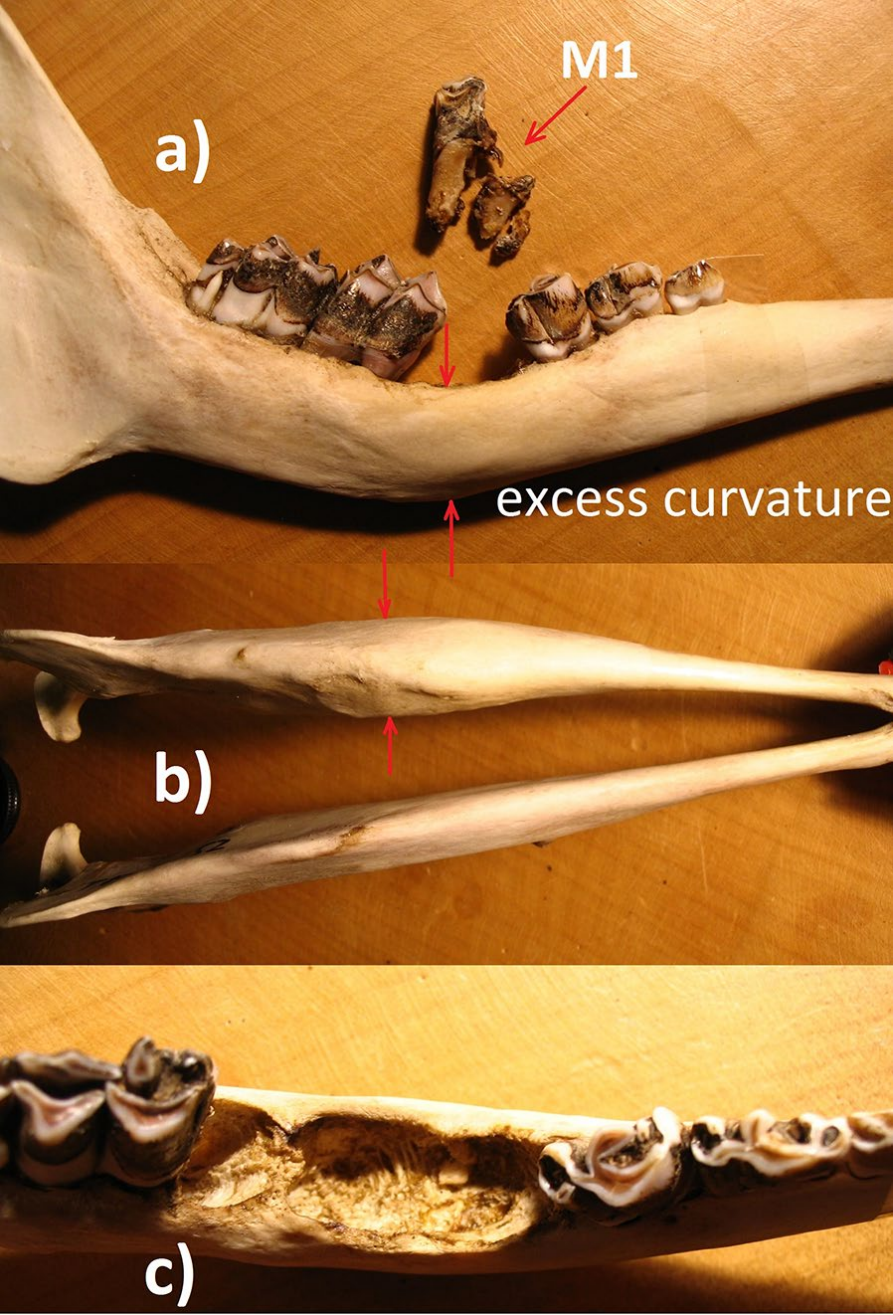
**a** Reduced height of the right mandibular body due to osteolysis with ventral border bent excessively during bone restructuring, fractured M1 with pieces merely held by soft gum tissue, misaligned M2; **b** thickened body of right mandibula at the level of molars M1 and M2 from necrosis and growth of porous bone matrix; **c** absent alveoli of M1 and M2 due to osteolysis

More severe pathology, however, is evident in the cranium. The right mandibular body shows height reduction from osteolysis (Fig. 2a), but elevated thickness (level of molars M1, M2, Fig. 2b), due to necrotic processes and extra growth of porous bone matrix. Alveoli had disappeared (Fig. 2c) and M1 is broken into several pieces that were merely held in place by soft tissue (Fig. 2a). As a result, M2 was misaligned and its alveoli also eliminated. Moreover, the ventral mandibular border bends excessively as a result of bone restructuring (Fig. 2a).

**Fig. 3:**
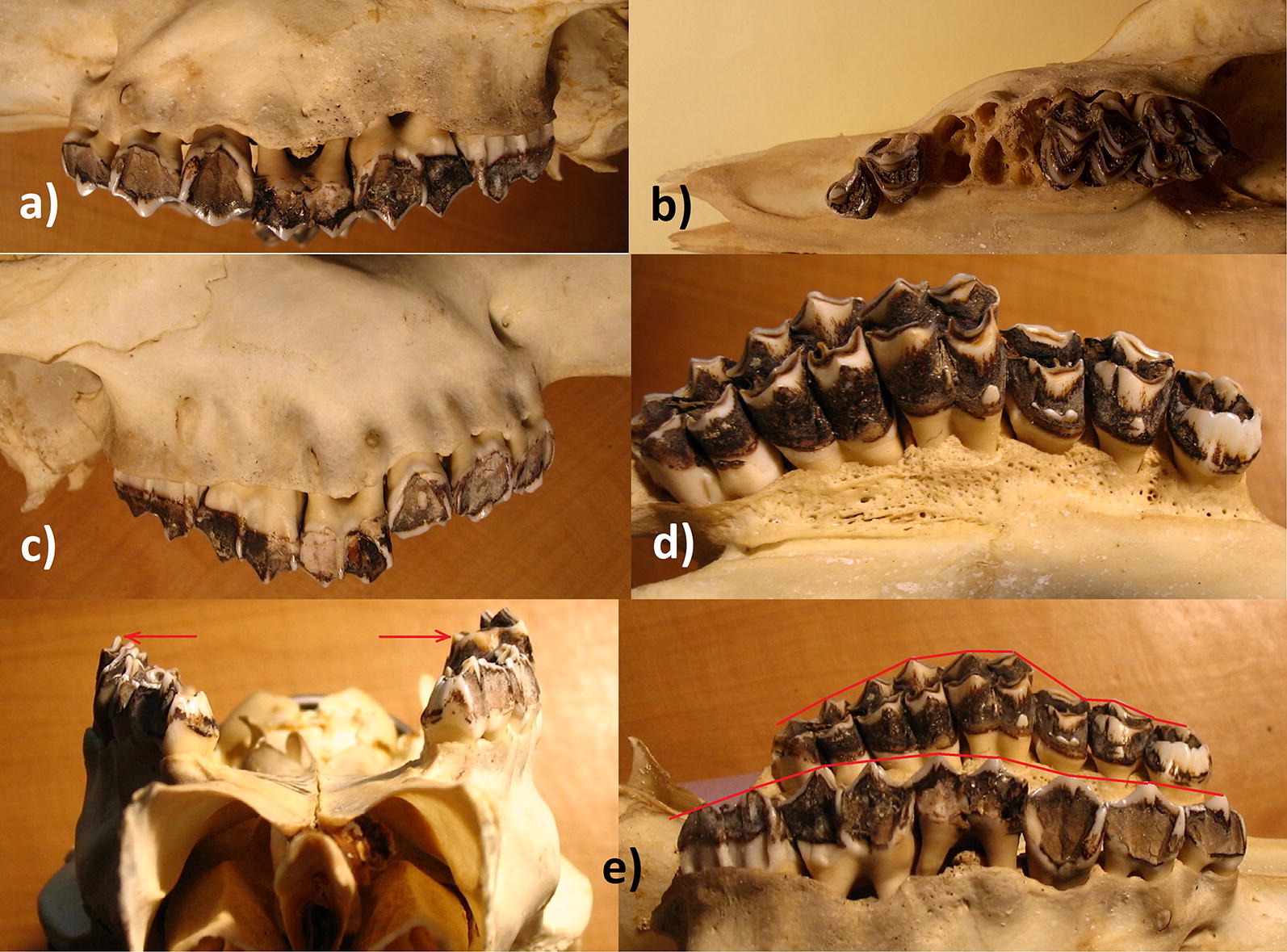
**a** Necrotic left maxillary, recessed and porous bone on the labial side, and exposed roots; **b** necrotic alveoli and exposed roots such that M1, M2 and P4 fall out in the absence of soft tissue; **c** necrotic right maxillary, recessed bone on the labial side including perforations with exposed roots; **d** the labial side with recessed and porous bone exposing dental roots; **e** the right maxillary molars protrude some 6 mm more than the left teeth row due to altered mandible

The maxillae are also very thin-walled such that dental roots are partially exposed. The left maxillary exhibits necrosis with resulting recessed bone on the labial side (Fig. 3a), necrotic alveoli, discolored roots, and porous maxillary walls indicative of inflammatory or even infective processes (Fig. 3b). M1 and M2 roots are exposed, and P4/M1 are so loose that they come out in the absence of soft tissue. The right maxillary exhibits necrosis, recessed bone on the labial side including perforations (fenestrations) which expose dental roots (Fig. 3c), and recessed and porous bone on the labial side with exposed roots (dehiscence; Fig. 3d). Lastly, due to aggravated pathological changes in the right mandible (fractured M1, displaced M2, Fig. 2a), the opposed maxillary molars had no resistance upon mastication and therefore, protrude approximately 6 mm more than the left tooth row (Fig. 3e), exhibit no wear for lack of contact, and bone restructuring on the lingual side resulted in porous bone matrix.

### Discussion

Endangered huemul (IUCN) [14] are characterized in Argentina by two decisive indices: the crucial demographics of very few 350–500 remaining animals (spread along 1850 km of Andes), and high prevalence of pathology in populations studied so far. One subpopulation (Protected Park Shoonem, Chubut province) had a prevalence of 57+ % of osteopathology among adults based on remains collected before 2007 [2], and recent evaluations of live adults in this population showed 86% affected with osteopathology, particularly cranial lesions [15]. Although prevalence has not been determined for other subpopulations, equivalent disease patterns occurred in other huemul 410 km further north, and in several individuals 275 km further south in Chile [16]. The present case, therefore, is unequivocal for this disease pattern from a new subpopulation 365 km further north (Additional file 1).

The pathophysiognomy reported here is similar to previous cases: thin-boned, perforations, bone resorption, exposed dental roots, and lose or displaced teeth. Similar pathological patterns were noted in wild ungulates in New Zealand, with worst cases occurring in areas of lowest selenium (Se) provision [17–19]. For cases from Park Shoonem, several primary etiologic factors were discarded for live huemul, for equal reasons as in earlier described dead cases [2] and instead, osteoarthritis and chronic alveolar osteomyelitis (secondary) in huemul were suggested to relate to nutritional ecology. Deficiency of Se in cervids and other ruminants impairs bone metabolism and causes periodontitis [23]: it occurs in huemul areas and is more prevalent where extant huemul tend to remain, namely at high altitudes. Such severe Se deficiency was corroborated in a Chilean huemul population, which simultaneously was also affected with this osteopathology [16, 20]. For southern Chile, the most common trace mineral imbalance recognized in dairy herds is Se deficiency [21]. The importance of Se stems from its role at very basic biochemical levels due to being coded genetically, and by forming part of selenocysteine: the recently discovered 21st naturally occurring amino acid [22]. Se deficiency reduces host defense, but moreover, also causes osteopenia and osteoarthritis by impairing bone metabolism [23–26]. Furthermore, Se deficiency has been documented in ruminants in similar environments elsewhere, and was recognized as underlying factor for mandibular thickening, periodontitis, premature loss of teeth, and bone density reduction [reviewed in 15], similarly to lesions in huemul [2, 15, 16, this paper]. For thyroid metabolism, Se is essential and therefore, Se deficiency is key for causing secondary deficiency of iodine [16]. Bone metabolism is known to be affected by both Se and iodine deficiencies, and this combined effect presents the most apprehensible explanation for absent recovery and various types of osteopathology dominating huemul populations. Importantly, deficiencies of iodine and Se also reduce recruitment and thus, the potential to colonize new areas, by having a strong direct impact on reproduction [10].

The biogeophysical situation of the case reported here is typical for extant huemul, being displaced from their natural traditional behavior to utilize low elevation habitat (as residents or via migrations). It coincides with huemul in this valley having been the most important food of early men, with concomitant practical absence of other animal species in diets [27, 28], and the resulting local extermination of huemul. Commonly, young bucks disperse substantial distances to establish new home ranges. This huemul, only 2.5 years old, which explains his appearance in the valley, likely as dispersor. Such behavior was recently corroborated after the first-ever reintroduction of huemul (Chile, 2016) [29]. However, with low-elevation and fertile areas generally settled by man, the few huemul dispersing no longer survive in these areas [10]. Populations keep diminishing without indications of recovery, likely due to effects on population dynamics via nutritional constraints in refuges where they remain.

Profound health conditions (as revealed here postmortem) demand that individuals be taken to rehabilitation centers. This is more urgent for severely endangered species because liberating such diseased animals would result in premature deaths and thus, important losses of reproductive lifetime. The ranch veterinarian providing xylazine had actually offered that the huemul be taken to their isolation enclosure specially made for cervids, but the authorities opted to resolve the situation in the capture site [30]. Yet the magnitude of osteopathology reported here qualifies as a severe disease stage. Several displaced teeth, fractured molar, loose teeth and secondary inflammations and possible infections result in debilitating pain during foraging and particularly during rumination. Ruminants with such dental problems will terminate in poor physical conditions, low reproductive rates, and low longevity, as documented for huemul previously [2, 15]. Hence, this young buck could have well served conservation efforts for many years if brought to an enclosure and kept under controlled conditions. It is striking, therefore, that the authorities subsequently examining this live buck, determined him to be healthy and apt for release [31]. Moreover, given his thus far good condition, his death most likely resulted from administering xylazine after having been tied to a post for 19 h, and absence of a reversal, which only allowed the slow metabolic breakdown.

Special attention must be given to the hitherto described pathophysiognomy among huemul [2, 15, 16, this paper], requiring thorough in vivo inspections of oral cavities. Valuable tools for in vivo evaluations of huemul possibly include infrared thermography which provides visual management tools for diagnosing, monitoring or treating injury, illness or disease [32, 33]. Thermography is non-invasive (Additional file 2) and does not require contact nor sedation, emits no radiation, can be repeated as frequently as required and can eliminate the need for other expensive investigations. Moreover, it allows detecting early lesions as described in this present case, even before they are clinically evident [34–37], and permits monitoring the healing process before the animal is released. Given the repeated evidence of osteopathology occurring in huemul over a broad geographical range and high prevalence among remains of dead and in live huemul [2, 15], it is essential that live huemul be examined utmost professionally.

## Limitations

The presented data is limited by sample size and by itself does not allow generalizations outside of the study area. Although infrared thermography has been a successful tool to diagnose diseases in other animal species, it has not yet been employed with huemul, and therefore, its applicability needs first to be confirmed.

## Additional files

**Additional file 1.** The various subpopulations of Huemul known to occur between 39° and 74°S (black squares, adopted from [10]), and those with documented cases of osteopathology (red squares).

**Additional file 2.** Healthy huemul buck: thermal images registered with a Testo T-890 camera.

## Abbreviation

Se: selenium
