# Supplementary figures and images for: Elusive cranial lesions severely afflicting young endangered Patagonian huemul deer
Source: BMC Res Notes. 2018 Sep 3;11:638. doi: 10.1186/s13104-018-3755-1 (PMC6122211; doi:10.1186/s13104-018-3755-1)

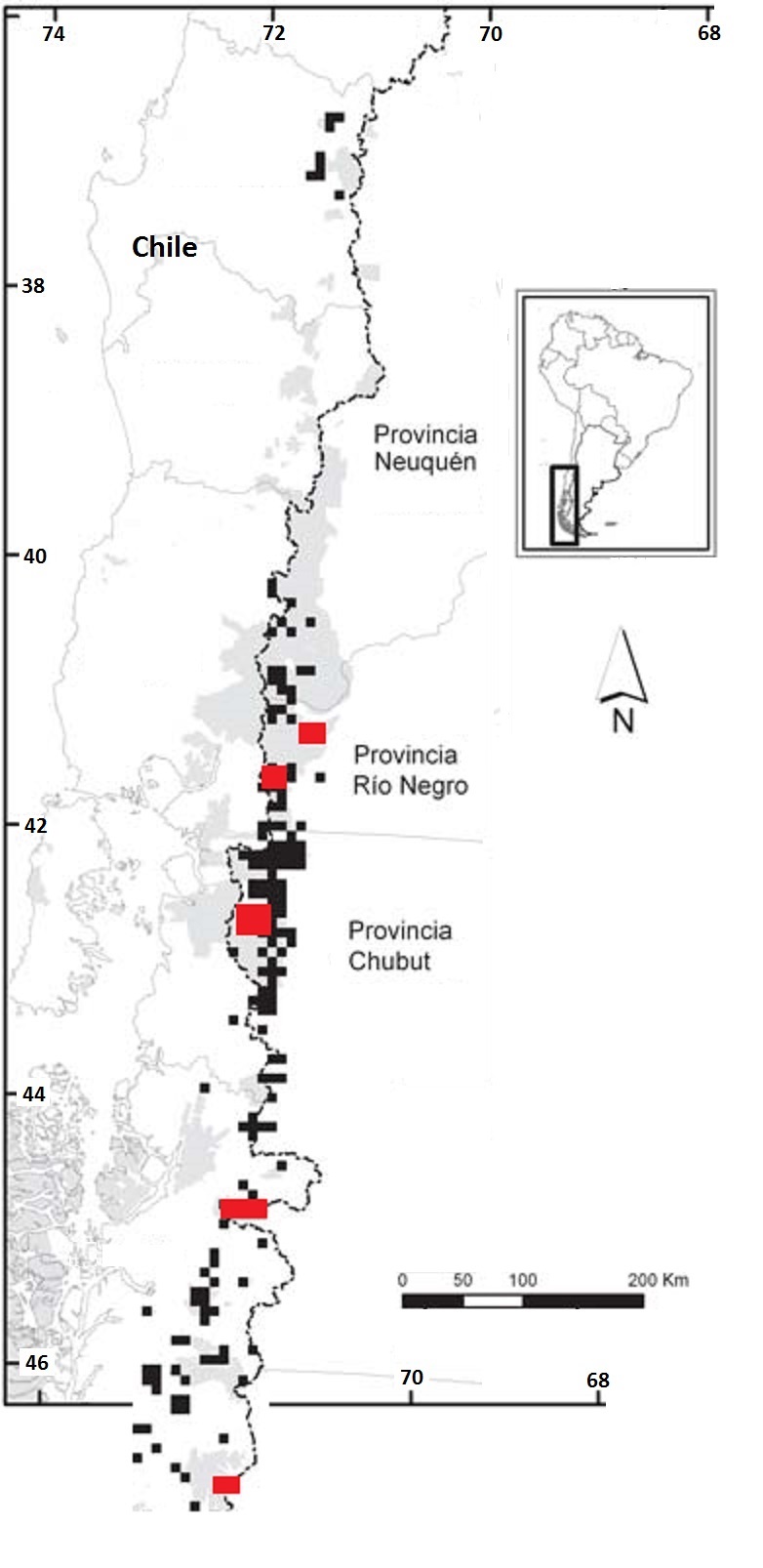

Supplement: Supplementary file 1 — Additional file 1. The various subpopulations of Huemul known to occur between 39° and 74°S (black squares, adopted from [10]), and those with documented cases of osteopathology (red squares). [file 13104_2018_3755_MOESM1_ESM.jpg]

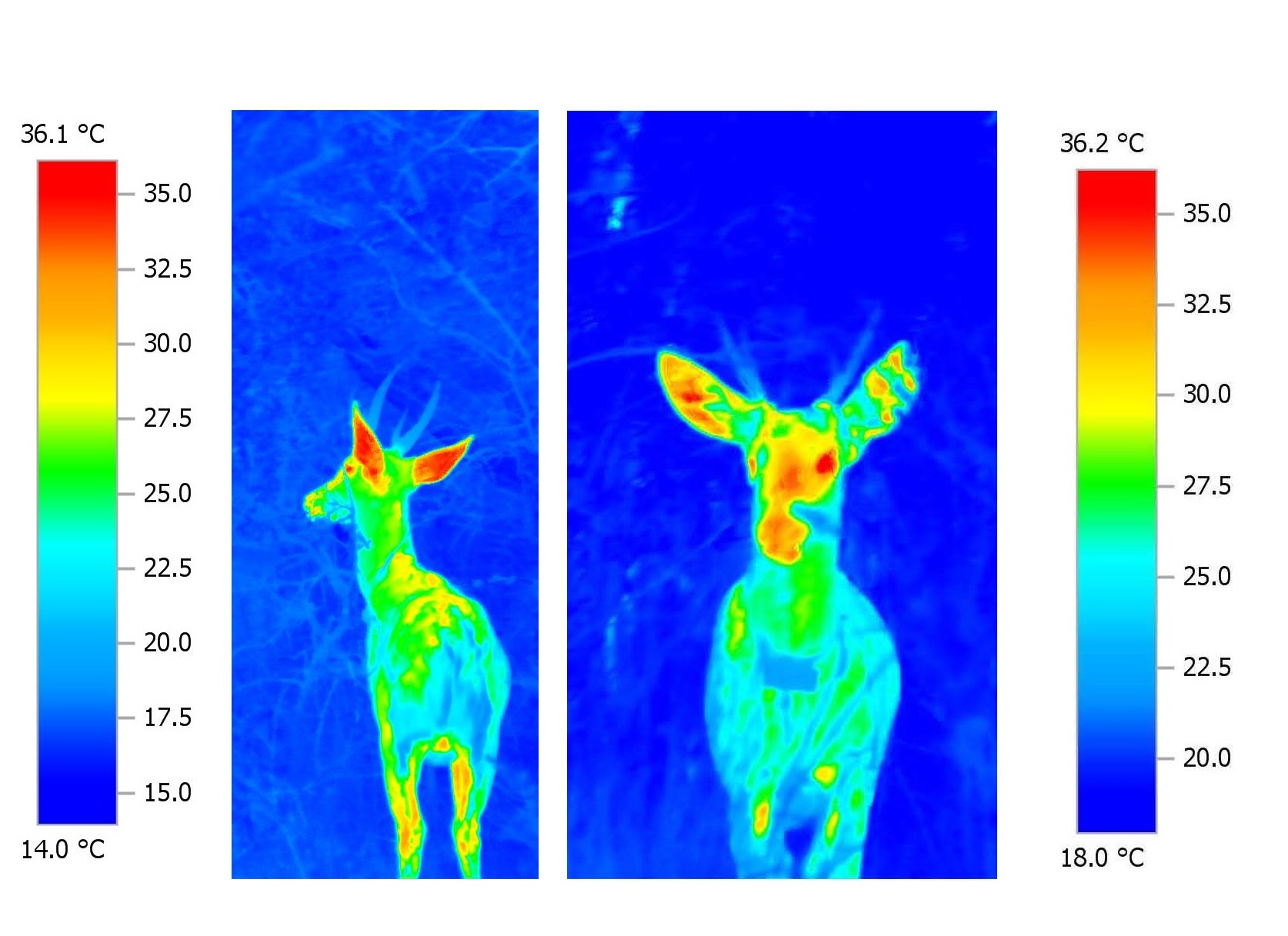

Supplement: Supplementary file 2 — Additional file 2. Healthy huemul buck: thermal images registered with a Testo T-890 camera. [file 13104_2018_3755_MOESM2_ESM.jpg]
